# Supplementary figures and images for: Optineurin deficiency impairs autophagy to cause interferon beta overproduction and increased survival of mice following viral infection
Source: PLoS One. 2023 Jun 23;18(6):e0287545. doi: 10.1371/journal.pone.0287545 (PMC10289332; doi:10.1371/journal.pone.0287545)

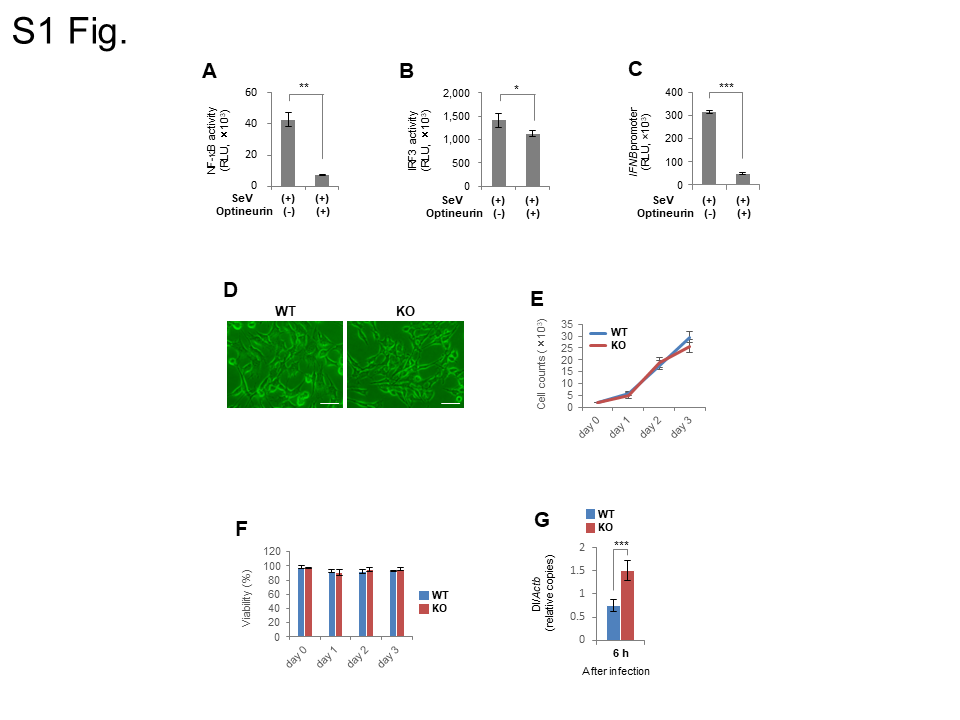

Supplement: S1 Fig — (A–C) Transcriptional activities of NF-κB (n = 3), IRF3 (n = 3), and the IFNB promoter (n = 3) in cells transfected with optineurin-expression or empty vectors 12 hours after viral infection of SeV (Cantell strain) were measured by luciferase assays. (D) Optn-KO and WT MEFs in steady state culture. Scale bars, 50 μm. (E and F) Cell growth and viability of Optn-KO and WT MEFs in steady state culture were measured by counting live and dead cells stained with trypan blue at the indicated days. n = 3 independent replicates per time point. (G) Relative viral DI genome copy numbers in WT (n = 4) and Optn-KO (n = 4) MEFs infected with SeV (Cantell strain) were measured by qPCR at 6 hours after inoculation. Data are presented as mean values ± SD. Two-tailed unpaired Welch’s t-test (A), two-tailed unpaired Student’s t-test (B, C, E, F, and G). *p < 0.05, **p < 0.01, ***p < 0.001. (TIF) [file pone.0287545.s001.TIF]

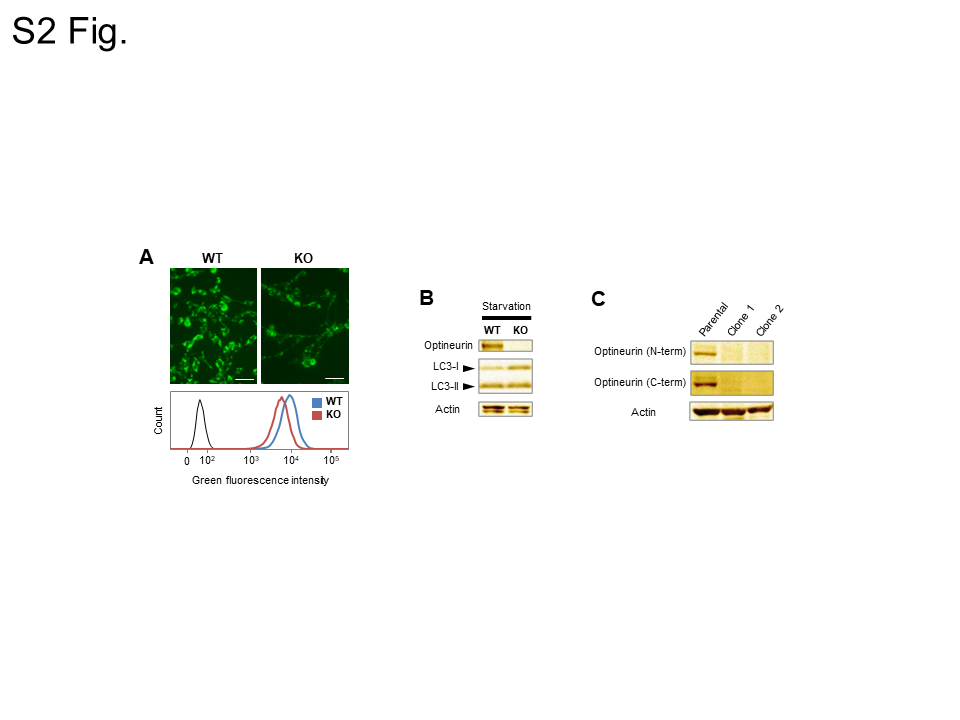

Supplement: S2 Fig — (A) (upper panels) Fluorescence images of WT and Optn-KO MEFs stained with the autophagy marker, CYTO-ID, under starvation conditions. Scale bars, 50 μm. (lower panel) Fluorescence intensities of WT and Optn-KO MEFs stained with CYTO-ID under starvation conditions were measured by flow cytometry and the results were compared. The black line indicates the unstained control. (B) LC3-I, LC3-II, optineurin, and actin of WT and Optn-KO MEFs under starvation conditions were examined by western blotting. (C) Optineurin protein levels in parental and two Optn-KO clones of GFP-LC3-RFP-LC3ΔG MEFs were examined by western blotting. (TIF) [file pone.0287545.s002.TIF]

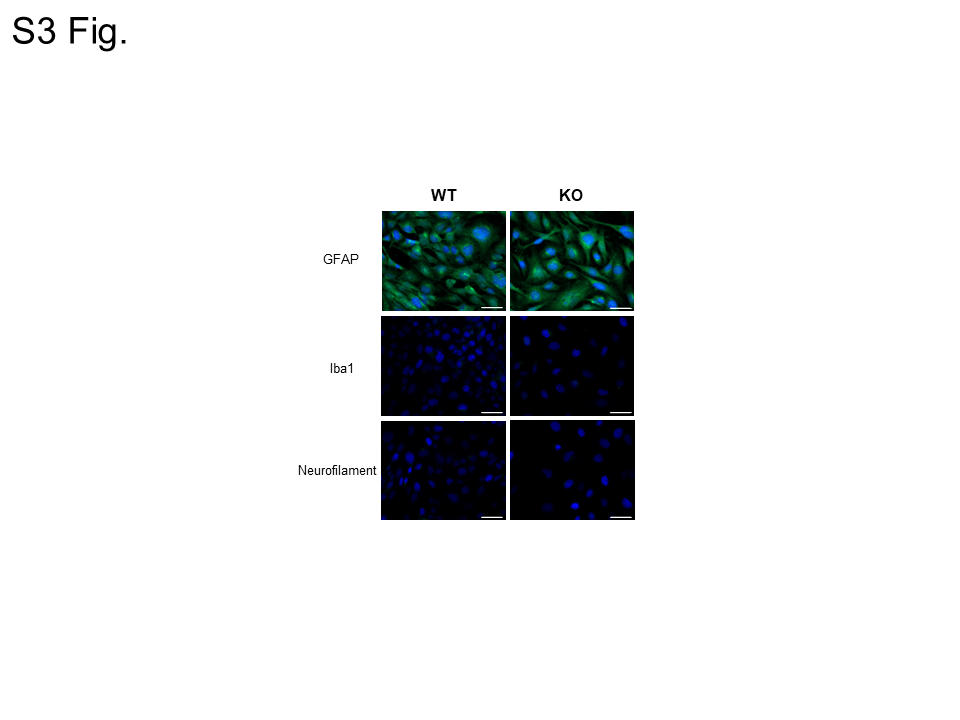

Supplement: S3 Fig — Fluorescence images of primary cells isolated from WT and Optn-KO mouse pups. The cells were stained with antibodies against the indicated proteins to confirm purity. Scale bars, 50 μm. (TIF) [file pone.0287545.s003.tif]

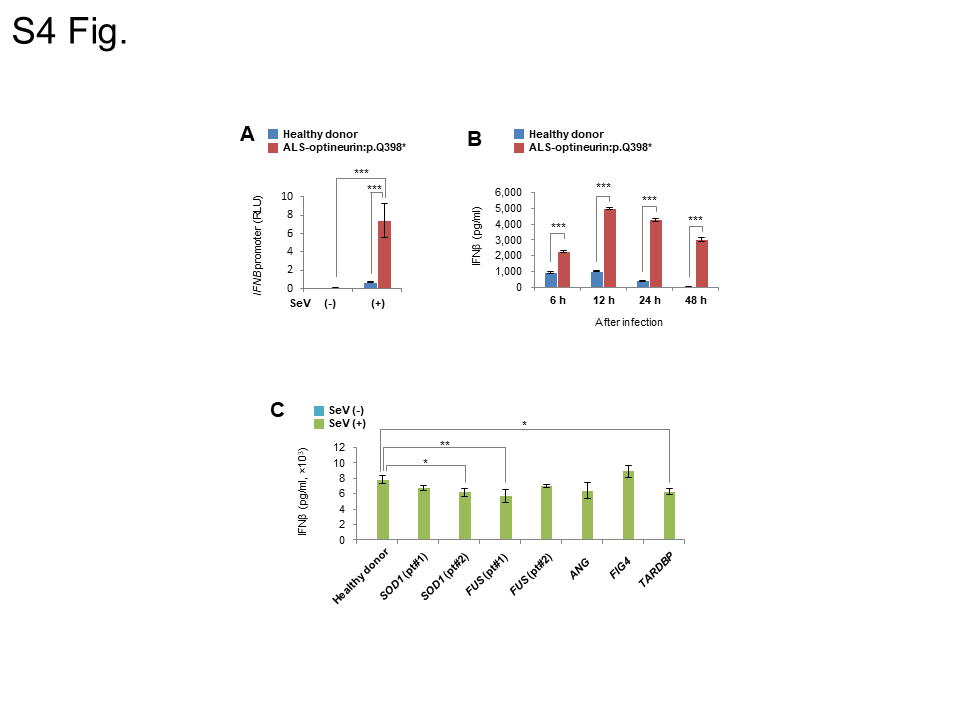

Supplement: S4 Fig — (A) Transcriptional activity of the IFNB promoter in healthy donor (n = 4) and ALS-optineurin:pQ398* patient (n = 4) fibroblasts was measured by luciferase assays at 24 hours after viral inoculation. (B) IFNβ production by healthy donor and ALS-optineurin:p.Q398* patient fibroblasts at the indicated hours after inoculation. n = 3 independent replicates per group at the indicated times. (C) IFNβ production from ALS patient fibroblasts carrying a mutation in the indicated causative genes (n = 3). The patient fibroblasts were infected with SeV (Cantell strain) or mock treated. Twenty-four hours after inoculation, IFNβ in culture medium was measured by ELISA. Data are presented as mean values ± SD. Two-way ANOVA followed by the Tukey–Kramer method (A and B) and one-way ANOVA followed by Dunnett’s test (C) were applied for statistical analyses. *p < 0.05, **p < 0.01, ***p < 0.001. (TIF) [file pone.0287545.s004.TIF]

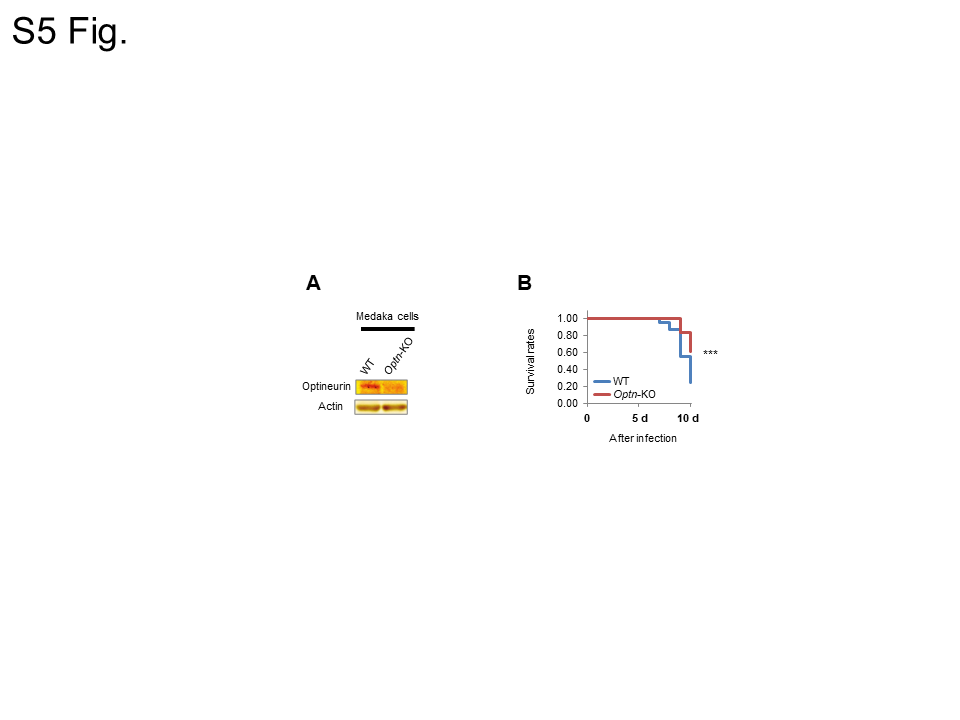

Supplement: S5 Fig — (A) Optineurin protein levels in cells isolated from WT and Optn-KO medaka (Oryzias latipes) were examined by western blotting. (B) Survival rates of WT (n = 60) and Optn-KO (n = 60) medaka infected with betanodavirus. The Kaplan–Meier method was applied for statistical analyses. ***p < 0.001. (TIF) [file pone.0287545.s005.TIF]

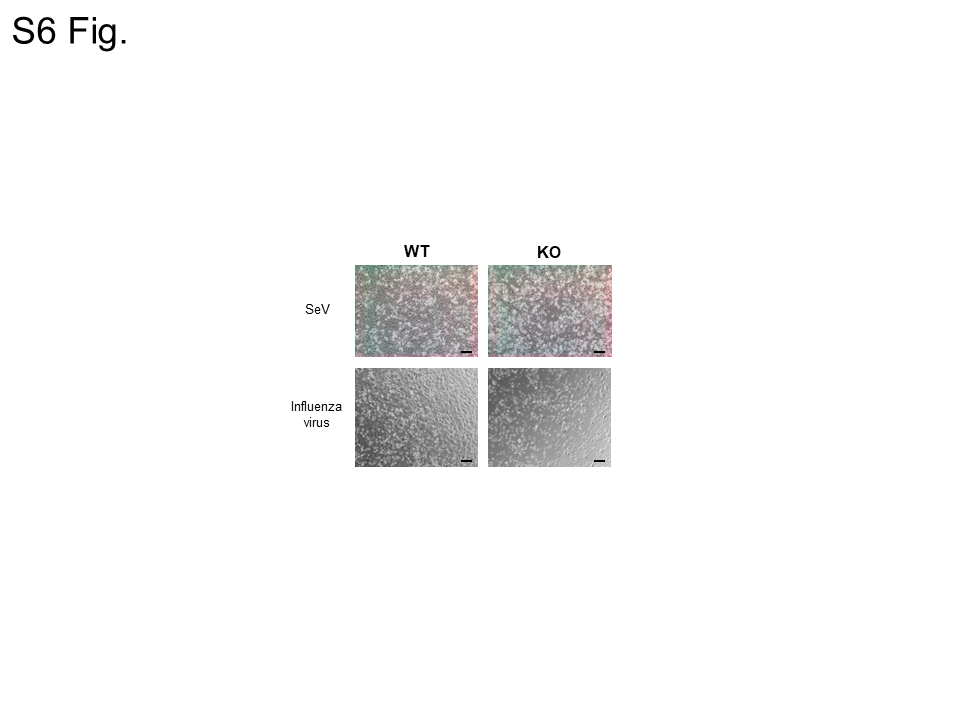

Supplement: S6 Fig — WT and Optn-KO MEFs infected with SeV (Z strain) 48 hours after inoculation and influenza virus (PR8 strain) 24 hours after inoculation. Scale bars, 100 mm. (TIF) [file pone.0287545.s006.TIF]

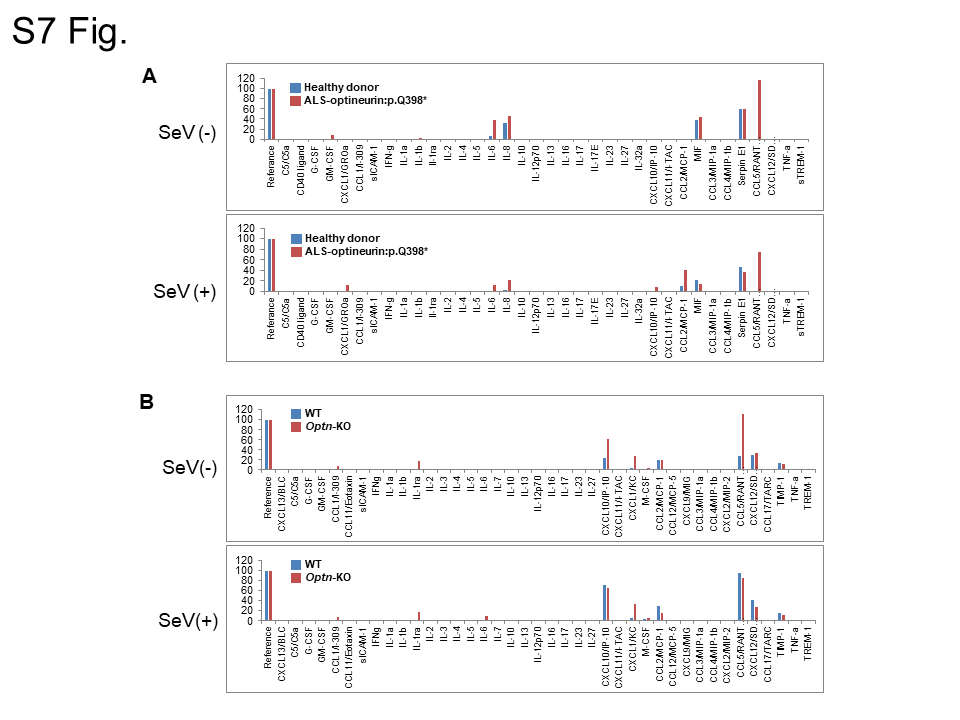

Supplement: S7 Fig — (A) Expression of 36 human cytokines by healthy donor and ALS-optineurin:p.Q398* patient fibroblasts infected with SeV (Cantell stain) or mock treated were examined by Proteome Profiler Antibody Arrays. (B) Expression of 40 mouse cytokines from WT and Optn-KO primary astrocytes infected with SeV (Cantell stain) or mock treated were examined by Proteome Profiler Antibody Arrays. (TIF) [file pone.0287545.s007.TIF]
