# Supplementary material for: Optineurin deficiency impairs autophagy to cause interferon beta overproduction and increased survival of mice following viral infection
Source: PLoS One. 2023 Jun 23;18(6):e0287545. doi: 10.1371/journal.pone.0287545 (PMC10289332; doi:10.1371/journal.pone.0287545)
Supplement: Attachment — Submitted filename: Response to Reviewers_v2 (20230529final).docx [file pone.0287545.s009.docx]

**I. Response to Additional Editor Comments**

Thank you for your valuable comments and suggestions. Our manuscript has been improved in response to the comments we received. Our point-by-point responses to each of the comments are listed below.

*1. Since several studies have reported findings contradictory to those presented in this manuscript, please include a discussion of those studies and possible reasons for the discrepancies.*

**Response:** Thank you for your comment. We have rewritten the Discussion section of the revised manuscript to describe the discrepancies between studies and provide possible explanations, as follows:

‘Regarding IFNβ production in optineurin-deficient cells, there are discrepancies between different reports (PMID: 24244017, 27086836, 26677802, 21862579, 20174559 and 25923723). We attribute these discrepancies to the cell types, stimuli, and methods used in the studies. In brief, some groups used bone marrow-derived macrophages (BMDM), LPS, and poly(I:C). LPS and poly(I:C) were directly added to the culture media of BMDM, which express Toll-like receptors (TLRs). Therefore, the stimuli of LPS and poly(I:C) are thought to be transmitted into cells through TLR 4 located on the cell surface or TLR 3 located in endosome. In our study, fibroblasts were mainly used that do not express TLRs, and viral infection was used as a stimulus. Viruses directly enter into the cytoplasm, and their genomes are recognised by cytoplasmic viral RNA sensors, such as retinoic acid-inducible gene I (RIG-I) and melanoma differentiation-associated protein 5 (MDA5), not by TLRs. Other groups also reported excess IFNβ production in optineurin-deficient fibroblasts during viral infection (PMID: 20174559 and 25923723). On the basis of these findings, we propose that the discrepancy in IFNβ production in optineurin-deficient cells is caused by a difference in the signalling pathways induced in response to stimuli.’

In addition, an *in vivo* study by Slowicka et al. (Eur. J. Immunol., 2016, PMID: 26677802) showed that mortality was slightly higher in OPTN knockout mice (4 of 7 mice died) compared with control mice (2 of 7 mice died). However, in their report, only 7 mice were used, and no statistically significant difference was found. By contrast, sufficient numbers of mice were used in our *in vivo* infection experiments of both SeV infection (*Optn*-KO, n = 40; WT, n = 39) and influenza virus infection (*Optn*-KO, n = 44; WT, n = 50), and significant differences were shown by statistical analysis. Therefore, we propose that the *in vivo* differences between our results and that of the other group may be a result of the number of animals used in the experiments.

*2. Please also provide information about the impact of the human Q398* mutation on protein function.*

**Response:** A previous study by our group showed that optineurin protein itself is not produced in ALS-optineurin:p.Q398* cells by a mechanism thought to be nonsense-mediated mRNA decay (Maruyama et al., Nature 2010, Supplementary Figure 3a, PMID: 20428114). Therefore, ALS-optineurin:p.Q398* cells are thought to show a similar response to that of *Optn*-KO cells.

**II. Response to the Reviewer #1’s comments**

*1. Authors need to show infectious viral titer during in vitro experiments*

**Response:** We apologize for any misunderstanding relating to our inadequate description of the SeV DI genome. The DI genome is specific to the Cantell strain of SeV and is not present in the SeV Z strain or influenza virus (Yoshida et al. J. Virology 2018, PMID: 29237838). It is also worth noting that the DI genome is replicated more rapidly than the full genome of SeV because it is shorter in length. IFNβ is known to be induced in response to viral infection. SeV Cantell strain is broadly used in studies of IFNβ production during viral infection because the DI genome of this strain is a strong inducer of IFNβ. However, IFNβ is also induced in response to infection by SeV Z strain that do not have DI genome in both *in vitro* and *in vivo* (Figs 1G and 5B). As pointed out by the reviewer’s comment, we were also interested in the viral titre of SeV. Therefore, we assessed the quantities of full viral genome in infected cells and the quantity was found to be higher in infected *Optn*-KO cells than in WT cells, similar to findings with the viral DI genome (Fig 1H).

We did not measure infectious viral titre of SeV in our *in vitro* experiments. This was because the measurement of infectious viral titre at 24 hours after inoculation was below the limit of detection. Instead, in this study, RT-qPCR was used to measure viral RNA levels in order to know viral replication (Fig 1H). In addition, *Optn*-KO cells showed more pronounced cell death compared with WT cells after 24 hours post-inoculation (S6 Fig). Therefore, we think that it is difficult to draw comparisons between the quantities of infectious virus particles produced by *Optn*-KO and WT cells after 24 hours post-inoculation. As shown in Fig 1H, both viral DI and full genomes were highly accumulated in *Optn*-KO cells. The reason of large quantities of both genomes in *Optn*-KO cells was shown to be caused by its low autophagic activity (Fig 2). However, we think that there is not necessarily a parallel between the large quantity of viral genomes inside the cells and the large quantity of infectious viral particles outside the cells. Because, from our *in vivo* study, the viral titre in *Optn*-KO mice was low, compared with WT mice. We speculate that the reason is that large amount of IFNβ strongly inhibits viral spread and also strongly leads to cell death of the infected cells.

*2. Authors need to show defective viral genome in vivo experiments.*

**Response:** In our mouse experiments, Z strain of SeV and influenza virus (PR8 strain) were used. Z strain and influenza virus do not possess a DI genome, as mentioned above. Therefore, we did not measure the quantity of DI genome in our mouse experiments. The reason why SeV Cantell strain was not used in *in vivo* experiments is that inoculation with the maximum titre of Cantell strain of SeV resulted in no change in the general condition of the animals, as mentioned in our manuscript. We showed that SeV Z strain also induced IFNβ overproduction *in vitro* and *in vivo* (Figs 1G and 5B). Therefore, IFNβ production is induced not only in response to DI genome but also full genome.

*3. This report makes a generalized claim that contradicts other published data regarding OPTN and viruses. The authors can only claim that this data is true for Sendai Virus (This reviewer 1’s comment is attached on top page of PDF file of the original manuscript).*

**Response:** Thank you for your comment. We have rewritten the Discussion section of the revised manuscript to describe the discrepancies between studies and provide possible explanations, as follows:

‘Regarding IFNβ production in optineurin-deficient cells, there are discrepancies between different reports (PMID: 24244017, 27086836, 26677802, 21862579, 20174559 and 25923723). We attribute these discrepancies to the cell types, stimuli, and methods used in the studies. In brief, some groups used bone marrow-derived macrophages (BMDM), LPS, and poly(I:C). LPS and poly(I:C) were directly added to the culture media of BMDM, which express Toll-like receptors (TLRs). Therefore, the stimuli of LPS and poly(I:C) are thought to be transmitted into cells through TLR 4 located on the cell surface or TLR 3 located in endosome. In our study, fibroblasts were mainly used that do not express TLRs, and viral infection was used as a stimulus. Viruses directly enter into the cytoplasm, and their genomes are recognised by cytoplasmic viral RNA sensors, such as retinoic acid-inducible gene I (RIG-I) and melanoma differentiation-associated protein 5 (MDA5), not by TLRs. Other groups also reported excess IFNβ production in optineurin-deficient fibroblasts during viral infection (PMID: 20174559 and 25923723). On the basis of these findings, we propose that the discrepancy in IFNβ production in optineurin-deficient cells is caused by a difference in the signalling pathways induced in response to stimuli.’

In addition, we used several viruses besides SeV Cantell and Z strains, including influenza virus and fish viruses. Therefore, the phenomena reported in this study do not appear to be limited to SeV infection.

*4. Please add Sendai and Influenza virus somewhere in the abstract (This reviewer 1’s comment is attached at line 56 on page 4 of the original manuscript).*

**Response:** We used several viruses besides SeV Cantell and Z strains, including influenza virus and fish viruses, as well as, several types of cells, and mice and fish. We have rewritten the Methods section of the Abstract in the revised manuscript to convey this.

*5. Show IFN B levels during poly I:C transfection (This reviewer 1’s comment is attached at line 331 on page 19 of the original manuscript).*

**Response:** Poly(I:C) is historically and broadly used as an IFNβ inducer instead of viral infection in virology and immunology fields. In this study, virus was infected to several types of optineurin-disrupted cells in order to measure IFNβ production. Because poly(I:C) transfection is more artificial than viral infection, we did not carry out a poly(I:C) transfection experiment for the measurement of IFNβ production. However, we speculate that IFNβ production in poly(I:C)-transfected *Optn*-KO MEF is greater than that of WT cells. In addition, Mankouri et al. (PLoS Path., 2010, PMID: 20174559) reported that optineurin-disrupted cells using siRNA produced excess IFNβ by poly(I:C) compared with WT cells. Therefore, to also avoid duplication, we did not perform poly(I:C) transfection to *Optn*-KO MEFs regarding IFNβ production.

*6. Please show viral titers in the cells through plaque assay. Defective viral genome is shown but not infectious titer (This reviewer 1’s comment is attached at line 345 on page 20 of the original manuscript).*

**Response:** We examined the quantity of SeV full genome by RT-qPCR to determine the viral titre in the infected cells, and the full genome quantity was higher in *Optn*-KO MEFs compared with WT cells, similar to the findings with DI genome (Fig 1H). Both the DI and full genomes of SeV are known to induce IFNβ production and, therefore, large amounts of DI and full genomes result in the excessive induction of IFNβ in *Optn*-KO MEFs.

*7. Please show the DI genome quantities in mouse lungs (This reviewer 1’s comment is attached at line 474 on page 27 of the original manuscript).*

**Response:** In our mouse experiments, Z strain of SeV and influenza virus (PR8 strain) were used. Z strain and influenza virus do not possess a DI genome, as mentioned above. Therefore, we did not measure the quantity of DI genome in our mouse experiments. The reason why SeV Cantell strain was not used in *in vivo* experiments is that inoculation with the maximum titre of Cantell strain of SeV resulted in no change in the general condition of animals, as mentioned in our manuscript. We showed that SeV Z strain also induced IFNβ overproduction *in vitro* and *in vivo* (Figs 1G and 5B). Therefore, IFNβ production is induced not only in response to DI genome but also full genome.

*8. Please explain if the defective viral genome is lower in OPTN KO cells. It is important to show that OPTN KO cells have higher defective viral genome but lower infectious viral titer (This reviewer 1’s comment is attached at line 476 on page 27 of the original manuscript).*

**Response:** In this study, the quantities of both DI and full genomes in *Optn*-KO cells were greater than those of WT cells (Fig 1H). These large quantities of both viral genomes induced excessive levels of IFNβ production, and IFNβ is known to cause apoptosis in infected cells (Tanaka et al., Genes Cells, 1998, PMID: 9581980; Taniguchi et al., Curr Opin Immunol., 2002, PMID: 11790540). In fact, we observed higher levels of cell death in *Optn*-KO cells than WT cells following viral infection (S6 Fig). Taken together with our *in vitro* and *in vivo* results, we conclude that the quantity of viral genomes in *Optn*-KO cells was greater than in WT cells (Fig 1H), and viral titres in the lungs of *Optn*-KO mice were lower than those of WT mice (Fig 5 E and F). In addition, IFNβ overproduction and pronounced cell death were observed in *Optn*-KO cells and mice. Large quantities of viral genomes in optineurin-deficient cells during viral infection induced IFNβ overproduction, and excess IFNβ significantly enhanced the death of infected cells and the anti-viral state of uninfected cell. As a consequence, this strongly suppressed the spread of the virus. Strong inhibition of viral spread led to the improved survival of infected *Optn*-KO animals.

**III. Response to the Reviewer #2’s comments**

*1. There is a discrepancy about the role of optineurin in innate immunity. Its role is highly debated in the context of innate immunity. While here, the results suggest that optineurin dampens IFNb production after viral infection through the autophagic degradation of viral nucleic acids, at least 5 previous studies (PMID: 24244017, 27086836, 27538435, 26677802, 21862579) have reported that optineurin is in contrast required for the signaling leading to the production of IFNb both in vitro and in vivo. How do the authors explain this? Based on their results, at the molecular level, do the authors observe an increased phosphorylation of TBK1 and IFR3 in optineurin deficient cells following viral infection?*

**Response:** Thank you for your comment. We have rewritten the Discussion section of the revised manuscript to describe the discrepancies between studies and provide possible explanations, as follows:

‘Regarding IFNβ production in optineurin-deficient cells, there are discrepancies between different reports (PMID: 24244017, 27086836, 26677802, 21862579, 20174559 and 25923723). We attribute these discrepancies to the cell types, stimuli, and methods used in the studies. In brief, some groups used bone marrow-derived macrophages (BMDM), LPS, and poly(I:C). LPS and poly(I:C) were directly added to the culture media of BMDM, which express Toll-like receptors (TLRs). Therefore, the stimuli of LPS and poly(I:C) are thought to be transmitted into cells through TLR 4 located on the cell surface or TLR 3 located in endosome. In our study, fibroblasts were mainly used that do not express TLRs, and viral infection was used as a stimulus. Viruses directly enter into the cytoplasm, and their genomes are recognised by cytoplasmic viral RNA sensors, such as retinoic acid-inducible gene I (RIG-I) and melanoma differentiation-associated protein 5 (MDA5), not by TLRs. Other groups also reported excess IFNβ production in optineurin-deficient fibroblasts during viral infection (PMID: 20174559 and 25923723). On the basis of these findings, we propose that the discrepancy in IFNβ production in optineurin-deficient cells is caused by a difference in the signalling pathways induced in response to stimuli.’

Although a part of the results showed by Pourcelot et al. (PMID: 27538435) that you indicated is also contrary to our results, we cannot find suitable reasons to this discrepancy except virus strains. However, Mankouri et al. (PLoS Path., 2010, PMID: 20174559) and Génin et al. (PLoS Path., 2015, PMID: 25923723) showed excess IFNβ production in optineurin-deficient fibroblasts during viral infection similar to our results.

In addition, an *in vivo* study by Slowicka et al. (Eur. J. Immunol., 2016, PMID: 26677802) showed that mortality was slightly higher in OPTN knockout mice (4 of 7 mice died) compared to control mice (2 of 7 mice died). However, in their report, only 7 mice were used, and no statistically significant difference was found. By contrast, sufficient numbers of mice were used in our *in vivo* infection experiments of both SeV infection (*Optn*-KO, n = 40; WT, n = 39) and influenza virus infection (*Optn*-KO, n = 44; WT, n = 50), and significant differences were shown by statistical analysis. Therefore, we propose that the *in vivo* differences between our results and that of the other group may be a result of the number of animals used in the experiments.

In our study, we did not determine whether TBK1 and IRF3 are phosphorylated in optineurin-deficient cells following viral infection. However, IRF3 activity in our luciferase assay was elevated in *Optn*-KO cells during viral infection compared with uninfected cells (Fig 1C). Therefore, we think that IRF3 is phosphorylated in *Optn*-KO cells during viral infection. In addition, IRF3 activity was inhibited in optineurin-overexpressed cells during SeV infection (S1 Fig B). From these results, we think that IRF3 is functional in *Optn*-KO cells, and that there is a relationship between optineurin and IRF3 activity.

*2. Moreover, the authors have also analyzed cells from patients with mutations in optineurin which is really different to cells fully deficient in optineurin since mutations may impair some of its function but not all the functions like a full knock down.*

**Response:** A previous study by our group showed that optineurin protein itself is not produced in ALS-optineurin:p.Q398* cells by a mechanism thought to be nonsense-mediated mRNA decay (Maruyama et al., Nature 2010, Supplementary Figure 3a, PMID: 20428114). Therefore, ALS-optineurin:p.Q398* cells are thought to show a similar response to that of *Optn*-KO cells.

*3. In fig 4C, why only Sqstm1+/- cells overproduces IFNb but not -/- cells?*

**Response:** We are also interested in this finding, but currently have no direct evidence to explain this. However, we have added a possible explanation to the Discussion section, as follows:

‘The reason why *Sqstm1*(˗/˗) cells do not overproduce IFNβ remains unclear. However, *Sqstm1*(˗/˗) cells readily induced cell death compared with *Sqstm1*(+/˗) cells during viral infection. Therefore, we speculate that *Sqstm1*(˗/˗) cells produce less IFNβ because cell death occurs more readily.’
